# Effects of EGFR driver mutations on pathologic regression in resectable locally advanced non-small cell lung cancer treated with neoadjuvant chemoradiation and completion surgery

**DOI:** 10.1259/bjr.20220763

**Published:** 2023-10-24

**Authors:** Sarit Appel, Jair Bar, Akram Saad, Edith Michelle Marom, Damien Urban, Amir Onn, Hadas Gantz-Sorotsky, Ran Yosef Kremer, Alon Ben-Nun, Marina Perelman, Efrat Ofek, Rinat Yacobi, Sameh Daher, Adi Rasco, Zvi Symon, Yaacov Richard Lawrence, Jeffrey Goldstein

**Affiliations:** 1 Department of Radiation Oncology, Chaim Sheba Medical Center, Tel- Hashomer, Israel; 2 Department of Medical Oncology, Chaim Sheba Medical Center, Tel-Hashomer, Israel; 3 Tel Aviv University the Sackler Faculty of Medicine, Tel Aviv, Israel; 4 Department of Radiology, Chaim Sheba Medical Center, Tel-Hashomer, Israel; 5 Department of Thoracic Surgery, Chaim Sheba Medical Center, Tel-Hashomer, Israel; 6 Department of Thoracic Surgery, Assuta Medical Center, Tel Aviv, Israel; 7 Department of Pathology, Chaim Sheba Medical Center, Tel-Hashomer, Israel; 8 Thoracic Cancer Unit Cancer Division, Rambam Health Care Campus, Haifa, Israel; 9 Department of Medical Oncology, Kaplan Medical Center, Rehovot, Israel; 10 Department of Radiation Oncology, Tel-Aviv Medical Center, Tel-Aviv, Israel

## Abstract

**Objective:**

We hypothesized that driver mutations in epidermal growth factor receptor (EGFR) are associated with decreased pathologic response to neoadjuvant chemoradiation (NA-ChRT) in locally advanced non-small cell lung cancer (LA-NSCLC).

**Methods:**

Patients with Stage IIB-IIIA NSCLC treated with NA-ChRT, completion surgery, and underwent molecular profile testing were identified in a lung cancer database. Pathologic response was quantified using: (i) major pathologic response (MPR), (ii) complete pathologic response (pCR), and (iii) mean residual viable tumor cells (MRTC). Two groups were formed based on the presence or absence of driver mutations. Clinical and pathological correlations between the groups were studied.

**Results:**

Forty-seven patients underwent tumor molecular profile testing, NA-ChRT, and completion surgery. Compared to the no-driver mutation group, the driver mutation group had lower MPR (23% *vs* 71%, *p* = 0.003), pCR (0% *vs* 26%, *p* = 0.02), and higher MRTC (43.4% *vs* 15.8%, *p* = 0.009). Univariate analysis showed an increased MPR rate for smokers, squamous cell histology, ChRT-surgery interval >65 days, and no-driver mutations. Multivariate analysis showed that only no-driver mutations (OR 0.39, *p* = 0.02) remained significant for MPR. PD-L1 status did not affect MPR. At 2 years, the driver mutation group had lower rates of local control (Hazard ration [HR] 0.67, *p* = 0.17) and disease-free survival (HR 0.5, *p* = 0.001). Overall survival was similar for both groups (HR = 1.04, *p* = 0.86).

**Conclusion:**

Following 60 Gray NA-ChRT, tumors with a driver mutation had lower MPR and pCR rates than tumors without a driver mutation. PD-L1 was not associated with tumor regression.

**Advances in knowledge:**

Patients with resectable LA-NSCLC and an EGFR driver mutation treated with neoadjuvant-ChRT and completion surgery have reduced pathologic regression, lower local control rates, and shorter disease-free survival than patients without a driver mutation. Evaluation of molecular testing should be introduced in LA-NSCLC intended for prognostication and treatment decisions.

## Introduction

Epidermal growth factor receptor (EGFR/ErbB1) driver mutations are valuable biomarkers for predicting response to tyrosine kinase inhibitors (TKIs) in patients with metastatic lung adenocarcinoma.^1,2^ There is indirect evidence suggesting that the presence of EGFR/ErbB1 driver mutations may also offer prognostic information for patients with locally advanced non-small cell lung cancer (LA-NSCLC) treated with chemoradiation (ChRT).^3^ Evaluating the effects of TKIs in patients with LA-NSCLC treated with ChRT alone has been limited since molecular profiling of the tumor prior to starting treatment has not been mandatory.^4,5^ A recent analysis of the PACIFIC trial showed that patients with unresectable LA-NSCLC treated with adjuvant durvalumab following ChRT had improved disease-free survival (DFS) and overall survival (OS), suggested that patients with EGFR mutations may benefit less from treatment with adjuvant durvalumab than patients without EGFR mutations.^6^ However, since EGFR mutations were present in only 6% of patients enrolled in this trial, these results should be interpreted cautiously. Currently, most patients with unresectable LA-NSCLC are treated with definitive ChRT followed by adjuvant durvalumab without first testing for the presence of driver mutations.^4–7^

Since patients with driver mutations may benefit from including targeted therapies into their therapeutic regime, there is growing interest in extending TKIs to patients with LA-NSCLC who harbor EGFR mutations. Prior studies investigating adjuvant cetuximab or erlotinib/gefitinib failed to improve OS in patients with LA-NSCLC and driver mutations.^8–10^ More recently, the phase III ADAURA trial showed the use of adjuvant osimertinib for 3 years in patients with completely resected, EGFR mutated, Stage IB-IIIA NSCLC was associated with improved DFS compared to treatment with placebo (90% *vs* 44%, *p* < 0.001). Despite the use of adjuvant platinum-based chemotherapy (ChT) in 26% of patients with Stage IB disease and 76% of patients with Stage II-IIIA disease in addition to osimertinib, the improvement in DFS associated with the use of osimertinib compels further investigation with targeted therapies.^11,12^ Currently, to improve outcomes in patients with EGFR mutations, the phase III NEOADUARA trial is evaluating the use of neoadjuvant osimertinib in addition to adjuvant osimertinib in patients with resectable NSCLC,^13^ and the phase III LAURA trial is enrolling patients with Stage III unresectable LA-NSCLC to evaluate the use of osimertinib following completion of definitive ChRT.^14^

Zens et al^15^ and other authors suggested that the amount of pathologic regression in surgical specimens following NA-ChT or NA-ChRT in LA-NSCLC is an important prognostic factor associated with improved DFS and OS.^16–19^ Pataer et al showed in 192 NSCLC patients who received NA-ChT followed by surgery that both surgical pathologic stage and the presence of a major pathologic regression (MPR, ≤10% viable tumor cells) were associated with improved OS and DFS. Other authors concluded the same, making MPR a strong prognosticator for OS and DFS in patients with NSCLC.^20–22^ We hypothesized that driver mutations are associated with reduced tumor regression in surgical specimens following NA-ChRT.

To evaluate the effect of driver mutations on pathologic regression, we utilized a unique cohort of patients with resectable LA-NSCLC treated according to our institutional protocol with NA-ChRT followed by completion surgery (references blinded). Our primary end point was pathologic regression. Secondary end points were local control (LC), DFS, and OS. We correlated both primary and secondary end points to the presence or absence of EGFR mutations, PD-L1 status, and rare mutations.

## Methods

### Patients

We conducted a retrospective analysis of a database to identify patients with resectable Stage IIB-III NSCLC treated with NA-ChRT followed by pre-planned completion surgery that underwent tumor molecular profile testing at Sheba Medical Center between 2012 and 2021. Exclusion criteria for this analysis were unresectable disease, local or distant progression of disease during or after completion of ChRT, absence of molecular profile testing, radiation dose <50 Gy or ≥72 Gray (Gy), or non-concurrent ChRT. We excluded patients who underwent salvage surgery for progressive disease from this analysis.

Variables collected included age, sex, smoking history (current, past-smoker, never-smoker), clinical stage, gross tumor volume (GTV), lymph node volume, histology (adenocarcinoma, squamous cell carcinoma, and other histology), and tumor location (upper, middle, or lower lobes). We defined total tumor volume as the combined GTV and lymph node volumes. Treatment variables collected were the type of NA-ChT administered, radiation dose, radiation technique, use of adaptive replanning during the treatment course, use of durvalumab prior to surgery, the time interval between completion of NA-ChRT and surgery, and type of surgical procedure.

### Molecular testing

We performed molecular testing to identify genetic alterations in tumor DNA; on the primary tumor or lymph nodes before starting ChRT, on lesions resected after ChRT and surgery, or on metastatic lesions. Kits used to determine the biomarkers changed during the time frame of this study, and based on the change in the standard testing used during different periods included EGFR testing using INFINITI^™^ Analyze (Auto Genomics, Carlsbad, CA)^23^ till 2018. After March 2018, Oncomine (Thermo Fisher Scientific, Waltham, MA), a next-generation sequencing test kit that analyzes for the presence of 22 genes simultaneously, was used to test for EGFR, ALK, ERBB2, ERBB4, FGFR1, FGFR2, FGFR3, MET, DDR2, KRAS, PIK3CA, BRAF, AKT1, PTEN, NRAS, MAP2K1, STK11, NOTCH1, CTNNB1, SMAD4, FBXW7 and TP53.^24,25^ Cases were assigned and analyzed according to the dominant genetic alteration, defined as the genetic alteration with the highest percentage of expression when several genetic alterations were detected.

PD-L1 expression was tested on archived tumor samples and measured by immunohistochemistry (Ventana Medical Systems, Oro Valley, AZ). The percentage of PD-L1 immunohistochemistry (IHC) staining was recorded from the pathologic specimens either before or after NA-ChRT and graded into three groups; negative (<1% positive staining), weakly positive (1–49%), and strongly positive (≥50%).^26^

### Treatment

Since 2012, the practice at Sheba Medical Center for resectable LA-NSCLC was to deliver NA-ChRT using platinum-based doublet ChT concomitantly with RT to a dose of 60 Gray (Gy), followed by restaging and then completion surgery for cases without local or distant progression. The NA-ChRT and surgical treatment protocols utilized at our center have been reported previously.^27^ Since 2016, RT delivery protocol used volumetric arc therapy (VMAT, Varian Inc. Palo Alto, CA), with daily image-guided radiation therapy (IGRT) using cone beam computerized tomography (CBCT). As shown previously, using VMAT or three-dimensional conformal radiation therapy (3DCRT) planning resulted in similar rates of pathologic regression,^28^ thus both techniques were included.

Since the introduction of durvalumab, patients with borderline resectable stage-III disease, whose extent of disease was initially considered borderline for surgery, were offered the option of receiving adjuvant durvalumab. After repeated CT imaging and reevaluation, the multidisciplinary team decided to discontinue the durvalumab and proceed with completion surgery.

### Pathologic response

**Figure 1.**
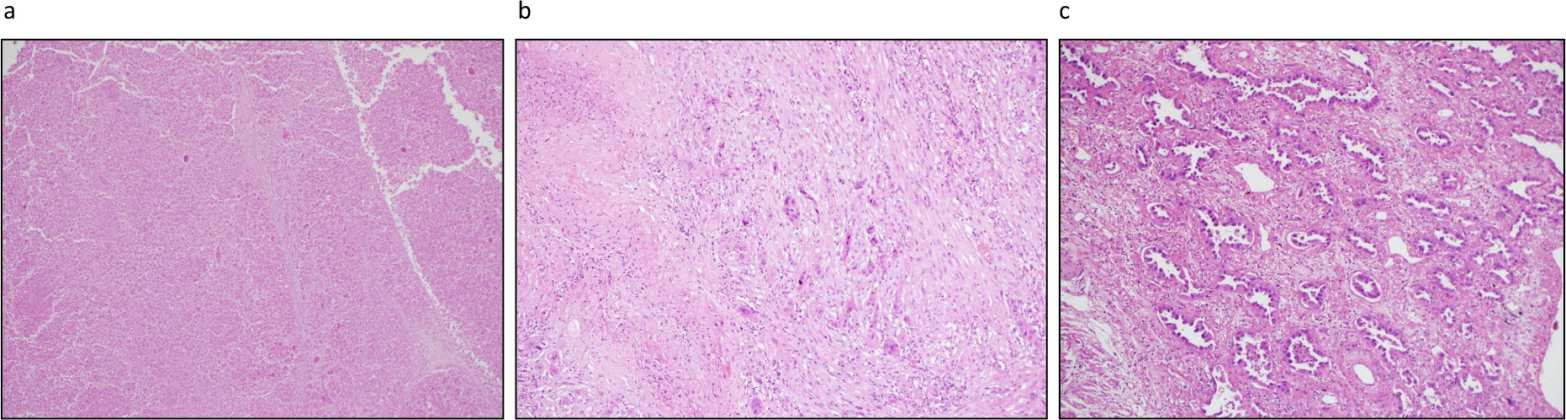
(a**–**c): Example pathology slides of lobectomies after chemoradiation showing treatment effect: (a) pCR pathologic compleate response, (b) MPR major pathologic regression and (c) Residual disease.

Pathologic response to NA-ChRT was assessed from surgical specimens and quantified using three measures of pathologic response: (i) major pathologic response (MPR ≤10% viable tumor cells) *vs* no MPR (>10% tumor viable tumor cells), (ii) complete pathologic responses (pCR) and (iii) the percentage of mean residual viable tumor cells (MRTC). The pathologists interpreting the specimens were unaware of the presence or absence of driver mutations. Figure 1 shows examples of treatment effects: pCR, MPR, and residual disease.

### Follow-up and clinical outcome

Patient follow-up examinations and imaging studies with CT or fludeoxyglucose (FDG) positron emission tomography (PET)-CT were done based on the standard of care, usually at 3 month intervals in the first-year post-surgery and then at 4–6 month intervals or as clinically indicated. We recorded the first recurrence as a local, regional, or distant failure.

### Statistical analysis

Descriptive statistics used to assess patient and treatment parameters included proportions, means, and standard deviations. We compared variables between groups using a χ^2^ test, unpaired *t*-test (for age), Wilcoxon rank-sum (Mann–Whitney) test, Kruskal–Wallis for continuous variables not normally distributed, and Spearman’s rank test to correlate MRTC with continuous variables. We conducted univariate and multivariate logistic regression to predict the odds ratio (OR) for MPR. Patients were sorted into groups based on the presence or absence of driver mutations and subdivided based on surgical specimens’ pathologic response. We excluded rare mutations represented by only a single case from the regression models. Multivariate analysis (MVA) models included variables with a *p*-value of < 0.2 in the univariate analysis (UVA). We excluded variables correlating with EGFR with significant (*p* ≤ 0.05) correlation coefficients from the MVA. OS was recorded from the start of NA-ChRT till death or censured at the last time the patient was known to be alive. Local and distant progression were recorded from patients' medical records or review of CT or FDG PET-CT imaging studies and was calculated from the initiation of NA-ChRT till progression or censure at the last evaluation. Time-to-event data were analyzed using the Kaplan–Meier method. We excluded driver mutations represented by a single case from the Kaplan–Meier analysis. We used hazard ratios (HRs) to compare local failure, distant failure, and survival rates. A *p*-value ≤ 0.05 was considered statistically significant. We used STATA v. 13 (StataCorp. 2013, Stata Statistical Software: Release 13. College Station, TX: StataCorp LP) for all statistical analysis.

## Results

During the study period, 107 patients with resectable LA-NSCLC were treated with NA-ChRT followed by surgery. Tumor molecular profile testing was available in 47 patients. Patients not undergoing testing for mutations (*n* = 60) were excluded from this analysis.

The patient characteristics, shown in Table 1, are subdivided into two groups based on tumor molecular profile testing results. The driver mutation group included patients with alterations in recognized drivers; EGFR (*n* = 13), BRAF (*n* = 1), RET (*n* = 1), and ROS1 (*n* = 1). The no-driver mutation group included all patients with no mutation identified (*n* = 17) as well as patients with mutation or aberration in FGFR (*n* = 2), KRAS (*n* = 10), ERBB4 (*n* = 1), or AKT (*n* = 1). Although some of these genes may be considered driver mutations, we designated this group the ‘no-driver-mutation group’ since, besides KRAS, none were proven drivers in lung cancer. Patients with KRAS mutation have clinical characteristics similar to patients with no-driver mutations, justifying their inclusion within the ‘no-driver mutation’ group.^29^

**Table 1.** Patient characteristics

Patient characteristic	All patients	No-driver mutation	Driver mutation	*p-* value
Patient number	47	31	16	
Age (mean, SD)	61 (8.9)	62 (8.6)	59 (9.4)	0.25
Sex:				0.58
Female	18	11	7	
Male	29	20	9	
Smoking:				0.00
Never	15	4	11	
Past	5	2	3	
Current	27	26	1	
Histology:				0.016
Adenocarcinoma	35	19	16	
Squamous cell carcinoma	5	5	0	
Other	7	7	0	
Stage (AJCC 7th ed.):				0.58
II	5	4	1	
III	41	26	15	
IV	1	1	0	
Tumor location:				0.8
Upper /middle lobes	34	22	12	
Lower lobes	13	9	4	
Chemotherapy:				0.14
Cisplatin-vinorelbine	10	7	3	
Carboplatin-paclitaxel	27	15	12	
Platinum-etoposide	7	7	0	
Cisplatin-pemetrexed	3	2	1	
Neoadjuvant durvalumab:				0.06
No	41	25	16	
Yes	6	6	0	
Surgery type:				0.25
Chest wall resection + lobectomy	4	4	0	
Lobectomy	34	22	12	
Pneumonectomy	8	5	3	
PDL1 IHC (total 34) percent (mean, (SD)	29.1(33)	29.4 (35)	28.7 (30.3)	0.95
Negative	11	9	2	
Weak positive (<50%)	12	6	6	0.24
Strong positive (≥50%)	11	8	3	
Radiation dose: (Mean, SD)	61 (5)	61.2 (5.5)	61 (5)	0.9
Planning technique:				0.26
three-dimensional fields (3DCRT)	20	15	5	
Volumetric arc (VMAT)	27	16	11	
Adaptive volumetric: replanning				0.13
Yes	10	9	1	
No	36	21	15	
Missing	0	1	0	
Total GTV volume primary + nodes (cm^3^), (mean, SD)	140 (119)	172 (132)	81 (54.5)	0.016
GTV, cm^3^, (mean, SD)	110 (123)	143 (137)	48 (47.5)	0.047
Lymph nodes volume, cm^3^ (mean, SD)	30.7 (32.6)	29.6 (37)	32.6 (22)	0.12
Lung V20 (%) (mean, SD)	23 (6)	23.1 (6.5)	23.4 (4.7)	0.76
Mean lung dose (Gy) (mean, SD)	13.6 (2.5)	13.6 (3.5)	13.6 (2.4)	0.83
Lung V5 (%) (mean, SD)	48.3 (14.6)	48.4 (16)	48.3 (11.6)	0.95
Heart V45 (%) (mean, SD)	6.5 (11.8)	7.9 (13.4)	4 (11.8)	0.4
Mean heart dose (Gy) (mean, SD)	9 (9.2)	9.65 (9.6)	8 (8.5)	0.7
Spine max dose (Gy) (mean, SD)	38.3 (11.7)	37.5 (13.2)	29.8 (8.3)	0.88
PTV D95% (mean, SD)	95.5 (3.1)	95 (2.7)	96.4 (3.7)	0.87
Interval between radiation and surgery (days) (mean, SD) (including durvalumab)	72.8 (44.7)	81 (50) (n=31)	56 (20)	0.01
Interval between radiation and surgery (days) (mean, SD) (excluding durvalumab)		61.8 (24) (n=25)	56 (20)	0.33
Interval between radiation and surgery (days) (mean, SD) (only durvalumab)		163 (44) (n=6)	56 (20)	0.002

Driver mutation group includes mutations in EGFR, RET, ROS and BRAF. GTV: gross tumor volume of the primary lesion, Lung V20: percent of lung receiving 20 Gray, Lung V5: percent of the lung receiving 5 Gray, Heart V45: percent of the heart receiving 45 Gray, PTV D95% the volume of the planning target volume receiving 95% of the dose. Programmed death-ligand one (PD-L1) Immunohistochemistry (IHC)

Comparison between the groups showed that the driver mutation group consisted of adenocarcinomas, mostly never smokers, had smaller GTVs (*p* = 0.047), smaller total tumor volumes (*p* = 0.016), and did not receive durvalumab post-ChRT (*p* = 0.08).

The no-driver mutation group had both adeno- and squamous cell carcinomas, were primarily smokers, had larger GTVs, larger total tumor volumes, and included a few patients (*n* = 6) who received durvalumab prior to undergoing completion surgery. The time interval between the end of NA-ChRT and surgery was longer for the six cases treated with durvalumab prior to surgery (mean 163 days, SD 44) than cases in the no-driver mutation group that did not receive durvalumab (mean 61.8 days, SD 24), or the driver mutation group (mean 56 days, SD 20, *p* = 0.002) (Table 1).

Table 2A presents each identified biomarker and its response to therapy measured by MPR, pCR, and MRTC in the surgical specimens following NA-ChRT. The driver mutation group demonstrated low pCR, MPR, and high MRTC. The MRTC for this group was 43.4% for EGFR (*n* = 13), 50% for RET (*n* = 1), 50% for ROS1 (50%, *n* = 1), and 45% for BRAF (*n* = 1). Mutations represented by only a solitary case each (RET/ROS1/BRAF) were excluded from the driver mutation group for the UVA and MVA, leaving an EGFR-only group (*n* = 13) for analysis.

**Table 2.** Pathologic response to chemo-radiation according to EGFR mutation

**Grouping**	**The leading mutation**		**MPR**			**pCR**			**Mean residual tumor cells (%)**	
Driver mutations	EGFR *N* = 13		3/13 (23%)			0/13 (0%)			43.4% (SD35)	
	BRAF *N* = 1		0/1 (0%)			0/1 (0%)			45%	
	RET *N* = 1		0/1 (0%)			0/1 (0%)			50%	
	ROS *N* = 1		0/1 (0%)			0/1 (0%)			50%	
No driver mutations	FGFR *N* = 2		2/2 (100%)			2/2 (100%)			0% (SD 0)	
	KRAS *N* = 10		8/10 (80%)			4/10 (40%)			13.7% (SD 23)	
	ERBB4 *N* = 1		1/1 (100%)			0/1 (100%)			5%	
	AKT amplification *N* = 1		1/1 (100%)			0/1 (100%)			9%	
	Negative for mutation *N* = 17		11/17 (64.7%)			3/17 (17.6%)			19.9% (SD24)	
**Biomarker grouping**		**MPR (n, %)**		***p*** **-value**	**pCR (n, %)**		***p*** **-value**	**MRTC (%, SD)**		***p*** **-value**
Driver mutations N = 16		3/16 (19%)		0.0001	0/16 (0%)		0.004	44.4% (SD 31)		0.001
EGFR only mutations *N* = 13		3/13 (23%)		0.003	0/13 (0%)		0.02	43.4% (SD 35)		0.009
No driver mutations *N* = 31		22/31 (71%)		reference	8/31 (26%)		reference	15.8% (SD 22.4)		reference

Abbreviations: Epidermal growth factor receptor (EGFR), Major pathologic response (MPR), Mean residual tumor cells (MRTC) Pathologic complete response (pCR). The driver mutations group include EGFR, RET, ROS1 and BRAF. The EGFR only group excluded RET, ROS and BRAF from the analysis. P values are for comparison of no- driver mutations to driver mutations and EGFR only mutations.

Mutations within the no-driver mutation group had higher rates of pCR, MPR, and lower MRTC. This group had low MRTC: 13.7% for KRAS (*n* = 10), 5% for ERBB4 (*n* = 1), 9% for AKT (*n* = 1), 0% for FGFR (*n* = 2), and 19.9% for patients who had no mutations identified (*n* = 17) on molecular testing. The no-driver mutation group was unchanged for the UVA and MVA. The no-driver mutation group was composed chiefly of cases with either KRAS mutation (*n* = 10) or no identifiable mutation (*n* = 17). These two subgroups of the no-driver-mutation group showed similar rates of pCR, MPR, and MRTC. Furthermore, Kaplan–Meier estimates of local control, DFS, and OS at 2 years for KRAS mutations and no-mutations showed no significant differences between cases with KRAS or no mutations identified (Figure S1 A–C). For KRAS *vs.* no mutations identified, LC at 2 years was 80% (95% CI 40–95) *vs.* 89% (95% CI 62–97) (*p* = 0.2), DFS was 60% (95% CI 25–83%) *vs.* 41% (95% CI 20–63%) (*p* = 0.7), and OS trended lower for KRAS: 70% (95% CI 33–89) *vs.* 90.5% (95% CI 67–97) (*p* = 0.18).

Supplementary Figure 1.

Comparison between the EGFR-only and no-driver mutation groups (Table 2B) showed the EGFR group had higher rates of MRTC (43.4% *vs.* 15.8%, *p* = 0.009) and lower rates of MPR (23% *vs.* 71%, *p* = 0.003) and pCR (0% *vs.* 26%, *p* = 0.02).

The UVA (Table 3) showed that the likelihood of MPR was increased for smokers (OR 0.34, *p* = 0.005), squamous cell histology (OR 0.53, *p* = 0.063), NA-ChRT surgery interval >65 days (OR 0.12, *p* = 0.005), and absence of an EGFR mutation (OR 0.35, *p* = 0.006). Cases receiving durvalumab before surgery trended towards a higher rate of MPR (83% *vs* 53%, OR 0.45, *p* = 0.16). PD-L1 status did not influence MPR.

**Table 3.** Uni- and multivariate analysis predicting the odds ratio for MPR

Variable	MPR N/TOTAL (%)	UVA		MVA	
		Odds ratio (95% CI)	*p*- value	Odds ratio (95% CI)	*p*- value
Age		0.99 (0.95-1.04)	0.78		
Sex:		0.5 (0.17-1.9)	0.34		
Female n=18	8/18 (44%)				
Male n=29	17/29 (58%)				
Smoking^a^:		0.34 (0.16-0.7)	0.004		
Never n=15	3/15 (20%)				
Past n=5	3/5 (60%)				
Current N=27	19/27 (70%)				
Histology^a^:		0.53 (0.3-1.01)	0.054		
Squamous n=5	5/5 (100%)				
Adenocarcinoma n=35	16/35 (46%)				
Other histology n=7	4/7 (57%)				
Stage:		0.3 (0.07-2.5)	0.35		
II n=5	2/5 (40%)				
III n=40	22/41 (53%)				
IV n=1	1/1 (100%)				
Tumor volume:		0.53 (0.16-1.7)	0.28		
≤115cc n=25	12/25 (48%)				
>115cc n=21	13/21 (62%)				
Location of primary tumor:		2.5 (0.6-9.8)	0.18	1.7 (0.4-7.5)	0.45
Upper lobe n=34	16/34 (48%)				
Lower lobe n=13	9/13 (69%)				
Chemotherapy:					
Cisplatin-vinorelbine	4/10 (40%)	0.98 (0.63-1.5)	0.92		
Carboplatin-paclitaxel Q1W	13/24 (54%)				
Carboplatin-paclitaxel Q3W	2/2 (100%)				
Cisplatin-etoposide	6/7 (86%)				
Cisplatin-pemetrexed	0/3 (0%)				
Durvalumab:		0.43 (0.15-1.3)	0.15	0.7 (0.2-2.1)	0.48
No n=41	20/41 (49%)				
Yes n=6	5/6 (83%)				
Radiation dose:		0.99 (0.3-3.4)	0.99		
≤55 Gy n=32	17/32 (53%)				
>56 Gy n=15	8/15 (53%)				
Radiation technique:		0.8 (0.25-2.5)	0.7		
3DCT n=20	10/20 (50%)				
VMAT n=27	15/27 (56%)				
Breathing management:		0.66 (0. 3-1.4)	0.3		
DIBH n=8	3/8 (37%)				
FB n=38	22/38 (58%)				
Adaptive planning due to tumor shrinkage:		2.9 (0.7-12)	0.13	1.6 (0.37-6.6)	0.53
Yes n=10	7/10 (70%)				
No n=36	17/36 (47%)				
No-EGFR n=31	22/31 (71%)	0.35 (0.16-0.7)	0.006	0.39 (0.18-0.86)	0.02
EGFR n=13	3/13 (23%)				
PDL1 status^b^:		1 (0.43-2.3)	1		
Negative n=11	6/11 (54%)				
Weak positive n=11	5/11 (45%)				
Strong positive n=11	6/11 (54%)				
Time interval radiation to surgery^a^:		0.12 (0.02-0.5)	0.005		
≤65 days n=30	11/30 (36.7%)				
>65 days n=17	14/17 (82%)				

Major pathologic regression (MPR), Gray (Gy), Three-dimensional conformal radiation therapy (3DCRT), Volumetric arc therapy (VMAT), Deep inspiratory breath hold (DIBH), Free breathing (FB), Epidermal growth factor receptor (EGFR), Chemoradiation (ChRT).

a Smoking, histology, and time interval between ChRT and surgery correlated with EGFR mutation (smoking *p* < 0.0001, histology *p* = 0.013, time interval between ChRT and surgery *p* = 0.048). Thus, they were excluded from the MVA.

b PDL1 status negative (< 1% positive staining); weak positive (1–49%) and strong positive (≥ 50%).

We excluded from the MVA the co-variables that correlated significantly with EGFR: never-smoking (*p* < 0.0001), adenocarcinoma histology (*p* = 0.013), and the time interval between radiation and surgery (*p* = 0.048). Only the no-driver mutation group (OR 0.39, *p* = 0.02) remained significant for tumor regression on MVA.

**Figure 2.**
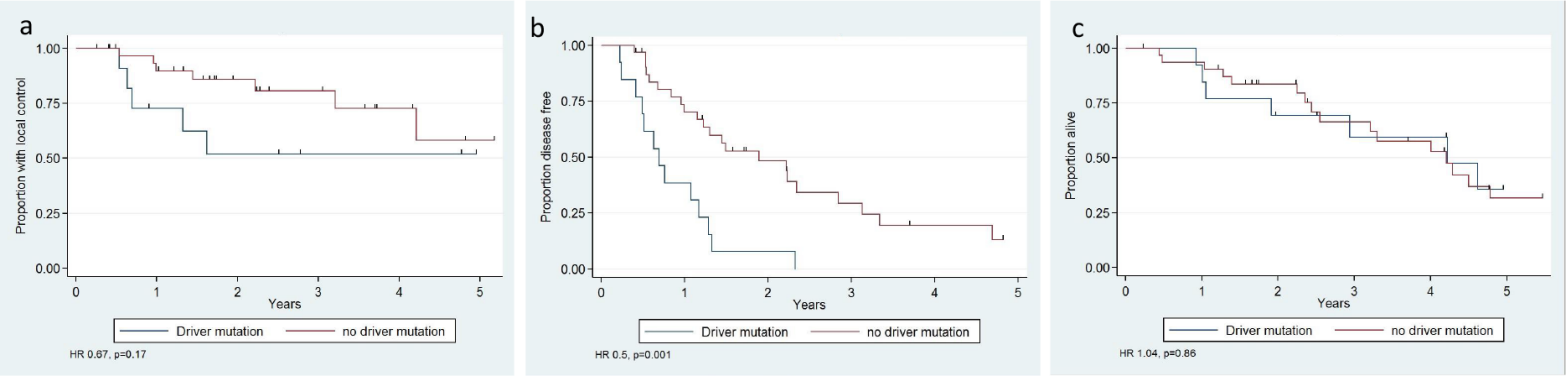
(a**–**c): Kaplan Meier estimated of (a) local control, (b) disease free survival and (c) overall survival for driver mutation (N=16) vs. no driver mutation (N=31).

Figure 2 compares local control, DFS, and OS at 2 years for the EGFR and no-driver mutation groups. 2-year DFS was lower for the EGFR group: 8% (95% CI 1–29%) *vs.* 45% (95% CI 29–65%), HR 0.5, *p* = 0.001. Local control trended lower for the EGFR group: 52% (95% CI 20–76%) *vs.* 85.8% (95% CI 66.5–95%), HR 0.67, *p* = 0.17. OS was not significantly different for the EGFR group: 69.2% (95% CI 37–87%) *vs.* 84% (95% CI 65–93%), HR 1.04, *p* = 0.86.

We knew PD-L1 receptor status in 37 cases. Table 4 presents the rates of MPR and pCR and the MRTC between negative, weakly positive, and strongly positive PD-L1 staining. There were no differences in MPR, pCR, or MRTC between the PD-L1 negative, weakly positive or strongly positive subgroups. Figure S2 presents a scatterplot showing MRTC according to the percent of PD-L1 staining, showing no correlation between the percentage of MRTC and the percentage of the PD-L1 positivity (*p* = 0.5).

Supplementary Figure 2.

**Table 4.** Pathologic response to chemoradiation according to and PDL-1 staining

PDL-1 staining	MPR (n, %)	*p*-value	pCR (n, %)	*p*-value	MRTC (%, SD)	*p*-value
Negative (*N* = 13)	7/13 (54%)	0.9	1/13 (7.7%)	0.56	22 (7.2)	0.56
Weak positive (*N* = 13)	6/13 (46%)		3/13 (23%)		29 (7.5)	
Strong positive (*N* = 11)	6/11 (54.5%)		3/11 (27%)		17.3 (7.8)	

Abbreviations: Major pathologic response (MPR), Mean residual tumor cells (MRTC), Pathologic complete response (pCR). PDL-1 negative <1% staining; weak positive 1-49% and strong positive ≥50%.

## Discussion

To the best of our knowledge, this is the first study in patients with LA-NSCLC describing pathologic regression as a function of EGFR mutation in surgical specimens following aggressive NA-ChRT using a radiation dose in the neoadjuvant setting similar to the doses used when delivering definitive treatment. We used three pathologic end points; the rate of MPR, the rate of pCR, and the percentage of MRTC to measure response to NA-ChRT. Comparison between the EGFR and the no-driver mutation groups showed lower rates of MPR (23% *vs.* 71%, *p* = 0.003) and pCR (0% *vs.* 26%, *p* = 0.02) and higher MRTC (43.4% *vs.* 15.8%, *p* = 0.009) in the EGFR mutation group. These three parameters all point to the same conclusion, the pathologic regression rates in surgical specimens following NA-ChRT were reduced if an EGFR mutation was present in the tumor.

In contrast to the poor outcomes reported in the EGFR mutation group, the pCR rate of 26% in our no-driver group was consistent with the rate of pCR reported in several large retrospective trials evaluating pathologic response following NA-ChRT in resectable LA-NSCLC. Lococo et al reported a pCR in 22% of cases in a study of 279 patients with LA-NSCLC treated with NA-ChRT,^30^ and Haque et al, in a large database study, reported the rate of pCR was 17%^31^ following NA-ChRT. In contrast to these findings, in our study, the rate of pCR in the EGFR group was 0% following an aggressive course of NA-ChRT that used higher RT doses than those used in these studies.

In addition to the poor response in surgical specimens, the DFS of patients with an EGFR mutation was also reduced compared to patients with no-driver mutations (HR 0.5 *p* = 0.001, Figure 2). Since the NA-ChRT protocol at our institution uses radiation doses commonly used when delivering definitive ChRT without resection, the low rates of pathologic regression and the short duration of DFS in patients with EGFR mutations observed in our study resonates with the poor results observed in patients with EGFR mutations treated with definitive ChRT. A recent study by Butkiewicz et al analyzed the outcome of 205 NSCLC patients treated with definitive platinum-based ChRT as a function of EGFR polymorphism (SNPs). They showed that patients with inoperable disease and functional EGFR genetic variants had poor clinical outcomes. They suggested that EGFR variants associated with elevated gene expression and protein activity may predict the decreased therapeutic efficacy of ChRT and that the EGFR SNPs may act as independent molecular prognostic predictors.^32^ Akimoto et al showed that the levels of EGFR expression correlated inversely with radiation-induced apoptosis, suggesting that lack of sensitivity to apoptosis induction may be an important mechanism responsible for the radioresistance of tumors with high EGFR expression.^33^ Chen et al described the role of EGFR in the repair of radiation-induced DNA damage. They suggested that EGFR confers tumor radiation resistance by activating survival and cell proliferation pathways.^34^ Further studies are required to gain insight into the role of EGFR and other driver mutations in RT-induced cancer cell death.

We found that adenocarcinoma and never-smoking status correlated with reduced tumor regression. These variables are known to be associated with EGFR mutations.^35,36^ Kayawake et al evaluated the response to ChRT in patients with NSCLC. They showed that patients with adenocarcinoma responded less to ChRT and that only squamous cell carcinoma was associated with achieving a pCR (*p* = 0.009).^3^ One of the differences between squamous cell and adenocarcinoma is that the latter is more likely to harbor a driver mutation such as EGFR, ALK, RET, or ROS1 which may explain the findings in Kayawake’s study.^37^

PD-L1 status did not correlate with pathologic regression measured by MPR, pCR, and MRTC. This finding contrasts with using the PD-L1 biomarker to predict response to immune checkpoint inhibitors in patients with metastatic disease.^37,38^ Combining treatment with an immune checkpoint inhibitor and radiation is under investigation and may improve response.^39^ Although durvalumab was used sparingly in this cohort, with only 6 of 31 patients in the no-driver mutation group receiving any durvalumab therapy, cases receiving durvalumab prior to surgery trended towards a higher chance for MPR (83% *vs.* 53%, OR 0.45, *p* = 0.16).

Patients with a KRAS mutation had more tumor regression in surgical specimens following ChRT and longer DFS following surgery than patients with an EGFR mutation, yet, trended towards a shorter duration of OS (Figure S1C). We suggest interpreting this finding cautiously due to the small number of patients in our study with KRAS mutations (*n* = 10). The discrepancy between the favorable rates of pathologic regression observed in our study and the poor prognosis reported for patients with KRAS mutations^29,40^ deserves further investigation. Enhancing treatment with immunotherapy or downstream inhibitors such as RAF or MEK inhibitors^41^ may improve clinical outcomes for these patients.

Potential bias in our data may result from selecting a single genetic alteration for analysis without accounting for other mutations that may co-exist and cause undetected interactions. This cohort included several genetic alterations. The rare mutations RET, ROS1, and BRAF were excluded from the EGFR group to produce a homogenous group for UVA and MVA regression analysis. We plan to investigate these rare mutations further in a larger cohort. Also, we had no cases of ALK rearrangement or MET mutation. Although the number of cases in the EGFR and no-driver mutation groups was small, the differences in pathologic response observed between the groups were sufficiently large to be significant. Other biases may result from using durvalumab in six patients within the no-driver mutation group. The improved outcomes with the use of neoadjuvant immunotherapy with chemotherapy compared to chemotherapy alone in patients with Stage IB-IIIA NSCLC reported recently in the Checkmate 816 trial^42^ suggests that the use of neoadjuvant durvalumab in six patients in the no-driver mutation group may have contributed to the better rates of pathologic regression and longer DFS observed in these patients. However, the proportion of patients who received durvalumab was small and unlikely to have impacted the results of MVA.

TKIs prolong survival significantly in patients with metastatic disease and EGFR-mutated tumors.^43^ Tyrosine kinase inhibitors were used in our cohort for recurrent disease, thus, explaining the discrepancy between shorter DFS yet similar OS between the driver mutation and the no-driver mutation groups (Figure 2). Since patients received TKIs only after surgery, their use did not affect the rates of pathologic regression observed in the surgical specimens.

For Stage IB-III NSCLC resectable EGFR mutated tumors, the ADUARA trial has shown that adjuvant osimertinib significantly prolonged DFS compared with placebo.^12^ Treatment with osimertinib has rapidly become the standard of care in these clinical settings. Current studies such as the NEOADAURA trial, which evaluates the use of neoadjuvant osimertinib in patients with resectable NSCLC prior to surgery,^13^ and the LAURA trial, which is investigating the use of adjuvant osimertinib following ChRT in patients with unresectable LA-NSCLC,^14^ may further improve outcomes in patients with EGFR mutated tumors treated with ChRT. Current guidelines for patients with unresectable NSCLC suggest treating patients with definitive ChRT and consolidation durvalumab regardless of driver mutation.^44^ As a result, deferring or not conducting molecular testing reduces the opportunity for patients to participate in clinical trials and potentially derive benefit from early treatment with targeted therapies such as osimertinib.^4,5^ Given the poor pathologic regression and short duration of DFS in the EGFR cohort detected in our study, we recommend that patients with LA-NSCLC undergo molecular testing for driver mutations allowing for targeted therapies to be introduced earlier in their course of treatment via clinical trials.

## Conclusions

Patients with resectable LA-NSCLC and a driver mutation had poor tumor regression in pathology specimens and shorter DFS following NA-ChRT and surgery. PD-L1 was not associated with tumor regression. Future studies in patients with LA-NSCLC should include molecular testing for tumor driver mutations for prognostication and treatment decisions.
