# Supplementary material for: Effects of EGFR driver mutations on pathologic regression in resectable locally advanced non-small cell lung cancer treated with neoadjuvant chemoradiation and completion surgery
Source: Br J Radiol. 2023 Oct 24;96(1152):20220763. doi: 10.1259/bjr.20220763 (PMC10646649; doi:10.1259/bjr.20220763)
Supplement: Supplementary Figure 1. [file bjr.20220763.suppl-01.pptx]

## Slide 1
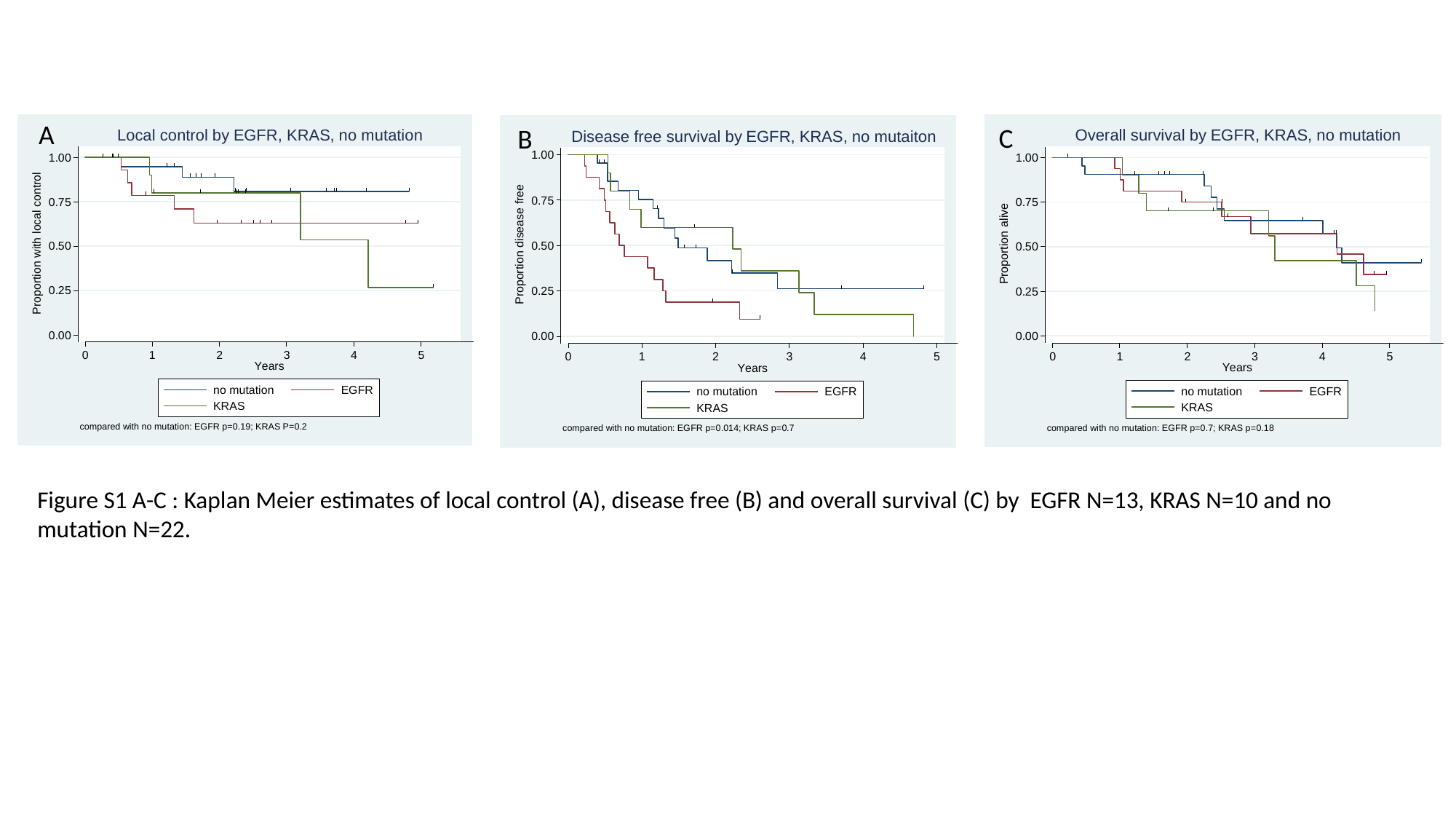

A
C
B
Figure S1 A-C : Kaplan Meier estimates of local control (A), disease free (B) and overall survival (C) by EGFR N=13, KRAS N=10 and no mutation N=22.
