# Supplementary figures and images for: Effects of EGFR driver mutations on pathologic regression in resectable locally advanced non-small cell lung cancer treated with neoadjuvant chemoradiation and completion surgery
Source: Br J Radiol. 2023 Oct 24;96(1152):20220763. doi: 10.1259/bjr.20220763 (PMC10646649; doi:10.1259/bjr.20220763)

## Slide 1
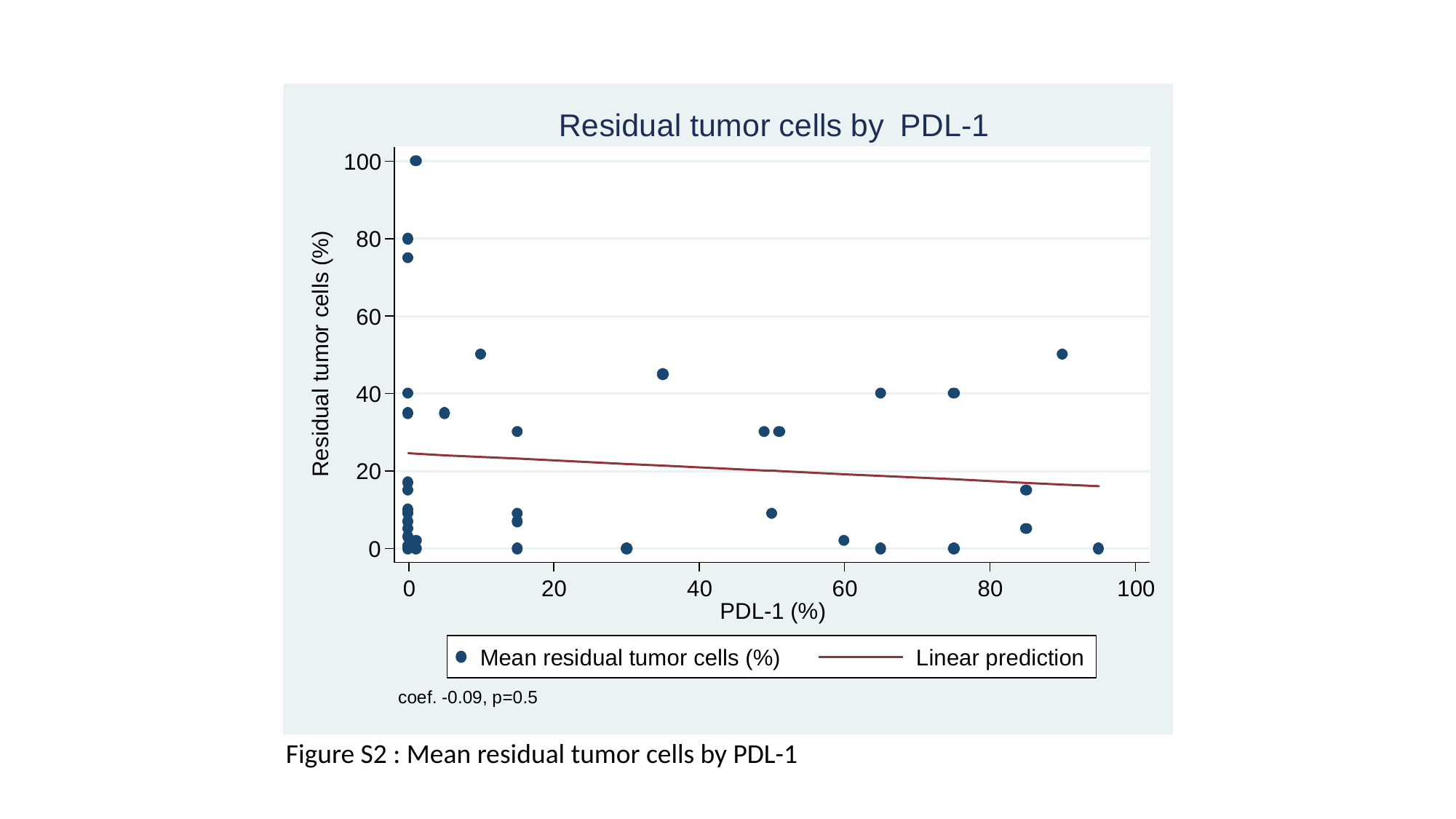

Figure S2 : Mean residual tumor cells by PDL-1

Supplement: Supplementary Figure 2. [file bjr.20220763.suppl-02.pptx]
